# A new species of *Hypoprepia* from the mountains of central Arizona (Lepidoptera, Erebidae, Arctiinae, Lithosiini)

**DOI:** 10.3897/zookeys.788.26885

**Published:** 2018-10-08

**Authors:** John Douglas Palting, Douglas C. Ferguson, Wendy Moore

**Affiliations:** 1 Graduate Interdisciplinary Program in Entomology and Insect Science, University of Arizona, Tucson, Arizona, 85721-0036, USA; 2 Systematic Entomology Laboratory, PSI, Agricultural Research Service, U.S. Department of Agriculture, c/o Smithsonian Institution, Washington, D.C., 20013-7012, USA; 3 Department of Entomology, University of Arizona, Forbes 410, Tucson, Arizona, 85721-0036, USA

**Keywords:** Lithosiini, Madrean fauna, mimicry, Sky Islands

## Abstract

A new firefly-mimicking lichen moth of the genus *Hypoprepia*, *H.lampyroides* Palting & Ferguson, **sp. n.**, is described from the mountains of east-central Arizona and the Sierra Madre Occidental of Mexico. *Hypoprepia* Hübner, 1831 is a North American genus of lithosiine tiger moths, previously consisting of five species: *H.fucosa* Hübner, 1831 and *H.miniata* (Kirby, 1837), both of eastern and central North America; *H.cadaverosa* Strecker, 1878 from the Rocky Mountains into New Mexico and west Texas; *H.inculta* H. Edwards, 1882, a widespread western USA species and *H.muelleri* Dyar, 1907 from the vicinity of Mexico City. The latter is herein synonymized under *H.inculta* (= *H.muelleri***syn. n.**), resulting in the total number of taxa in the genus unchanged at five.

## Introduction

The mountains of southeastern Arizona and northeastern Sonora are well known as a biological blending zone between the fauna of the Rocky Mountains to the north and Mexico’s Sierra Madre Occidental to the south. Positioned between these two great mountain ranges, the Sky Island Region contains a series of smaller mountain ranges that have oak and pine at higher elevations, each range being separated from one another by expanses of drier grasslands and desert. Sky Island ranges often harbor relict populations of plants and animals that suggest that in the distant past, both geology and climate allowed connections between the flora and fauna of the Rockies and the Sierra Madre (Warshall 1995). Examples among Lepidoptera of this connection include *Chiricahuamultidentata* (Guedet, 1941) and *Chiricahualichenaria* Ferris, 2010 (Geometridae, Ennominae), known in the US only from the highest elevations of the Chiricahua Mountains in SE Arizona, with the next nearest recorded population being in El Salto, Durango, nearly 900 miles to the south. A similarly striking disjunct population occurs with *Nemoriasplendidaria* (Grossbeck, 1910) (Geometridae, Geometrinae) known only from the top of the Huachuca Mountains, Arizona in the US with the nearest Mexico records also being from Durango. *Alexiclesaspersa* Grote, 1883 (Erebidae, Arctiinae) occurs sporadically from Colorado to several places in the White Mountains of central Arizona, adjacent parts of New Mexico, and not again until the top of the Sierra Madre in the vicinity of Yecora, Sonora, Mexico, skipping the Sky Island ranges entirely. Other rare US Lepidoptera that exhibit similar but less dramatically disjunct distributions include the lasiocampids *Caloeciaentima* Franclemont, 1973 and *C.juvenalis* (Barnes & McDunnough, 1911) (Lasiocampidae, Lasiocampinae), *C.entima* known in the US only from the high elevations of the Chiricahuas, and *C.juvenalis* only from the Chiricahuas and Huachucas, with spotty distributions in the Mexican state of Sonora (Sierra Mariquita, Sierra del Tigre and Yecora). *Agyllaseptentrionalis* Barnes & McDunnough, 1911 (Erebidae, Arctiinae, Lithosiini) is also known from isolated high-elevation populations in the Chiricahua and Huachuca Mountains, separated from the nearest Sierra Madre populations in Yecora, Sonora by 400 miles. These are just a few of many examples among Lepidoptera species with relict disjunct distributions indicating an historical Rocky Mountain-Madrean connection in this region.

We can now add another rare species of Lepidoptera from Arizona to the body of evidence supporting this past faunal connectivity. The moth was first noticed by the late Ron Leuschner, who collected a specimen on the door of a rental cabin in the hamlet of Greer, White Mountains, Arizona in 1988. Leuschner sent this specimen to Ferguson, who, prior to his death in 2002, recognized it as new and started to describe it based upon this specimen and two additional specimens he located in collections. Ferguson had dissected and made some comments on the male genitalia, but had not examined the internal structures of the female.

**Figures 1–2.**
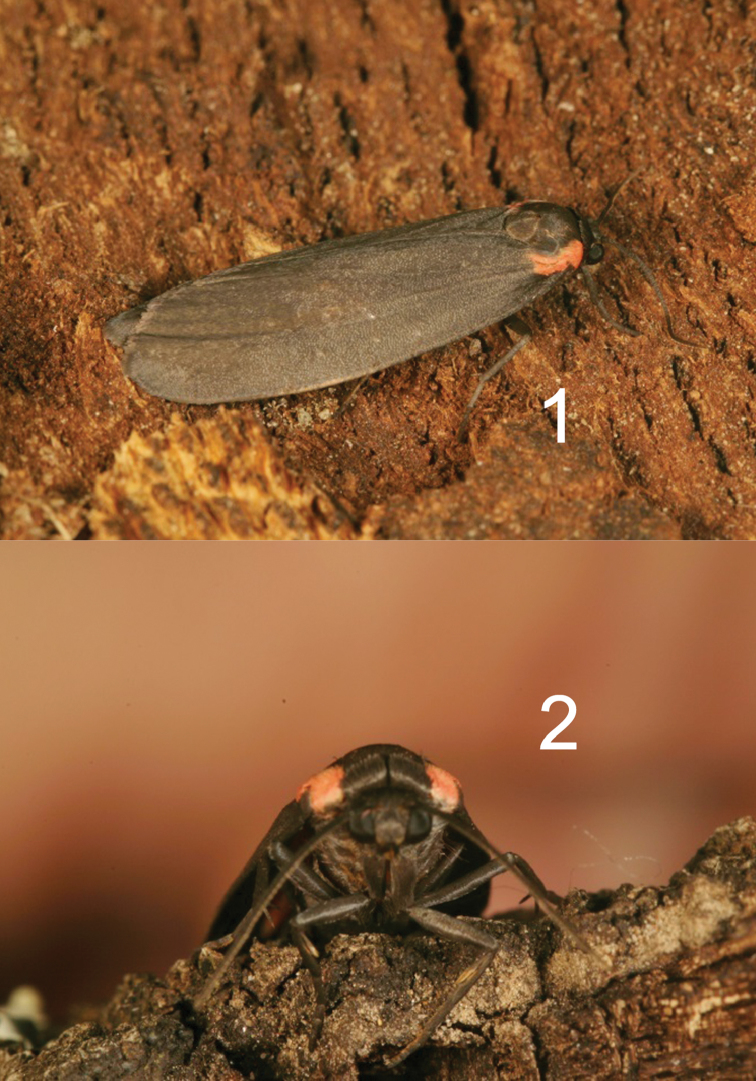
Two views of living male *Hypoprepialampyroides*.

**Figures 3–4.**
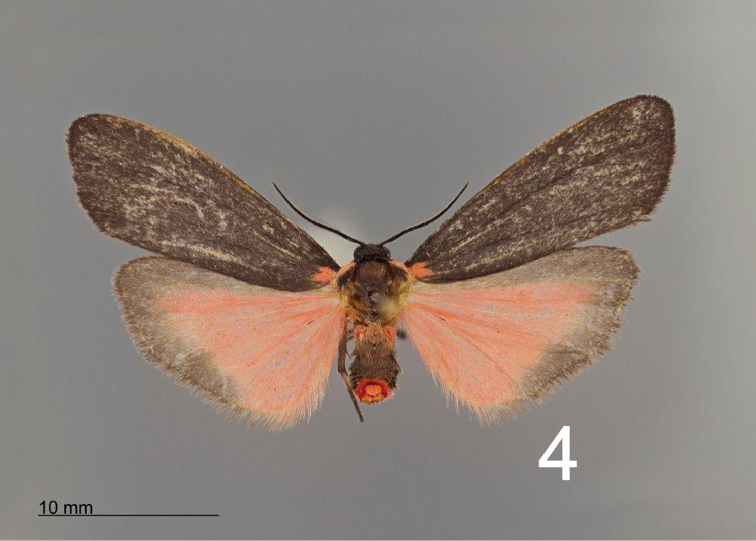
Adults of *Hypoprepialampyroides*. **3** male and **4** female.

**Figures 5–6.**
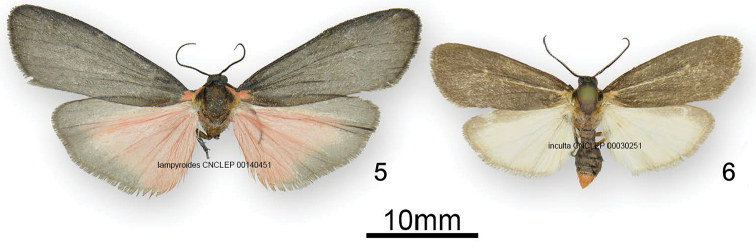
Adult male **5***Hypoprepialampyroides* and **6***Hypoprepiainculta*.

In June 2017, JDP and Ray Nagle had the good fortune of collecting more than 30 specimens of this new species along Highway 191 in the vicinity of Rose Peak, Blue Ridge Primitive Area, Greenlee County, Arizona. Flying sympatrically with *Hypoprepiainculta* Edwards, 1882 was the similar-looking, but much larger bodied and more boldly colored, *H.inculta* look-alike (Figs 1–5). Finally, here was the almost mythical moth that Leuschner had found nearly 30 years prior in Greer. Its similarity to *H.inculta* (Figure 6), combined with narrow endemicity and an early flight period just prior to or at the onset of the summer rains, may account for the paucity of records of this new species. It appears to fly throughout the night, with new individuals showing up on the sheet with regularity until dawn, outnumbered by *H.inculta* by approximately 4 : 1. Most of the specimens collected were males, but two females of the new species were collected and kept alive for ova, allowing for the larvae to be reared and photographed for the first time.

Other noteworthy species flying alongside the *Hypoprepia* were *Nadatagibbosa* (JE Smith, 1797) (Notodontidae, Phalerinae) and *Spilosomavirginica* (Fabricius, 1798) (Erebidae, Arctiinae, Arctiini), both common northern and eastern species, but at the extreme southern limit of their ranges here, as well as *Apantesisf-pallida* (Strecker, 1878) (Erebidae, Arctiinae, Arctiini), a primarily Rocky Mountain species, very rare this far southwest. Also present was the strikingly beautiful *Erastriaviridiruferia* (Neumoegen, 1881) (Geometridae, Ennominae), another Madrean species that occurs in central Arizona, with sporadic records from the Sky Islands Region through the Sierra Madre proper, where it occurs regularly at mid to high elevations.

## Methods and materials

### Phylogenetic analysis

Total genomic DNA was extracted from the right middle leg of each voucher specimen using the Qiagen DNeasy Blood and Tissue Kit (Qiagen, Valencia, CA), according to manufacturer suggested protocol. The barcoding region of the mitochondrial gene cytochrome oxidase subunit 1 (COI) was PCR amplified with primers LCO1490 and HCO2198 (Hebert et al. 2003). PCR products were cleaned, quantified, normalized, and sequenced in both directions at the University of Arizona’s Genomic and Technology Core Facility using a 3730 or 3730XL Applied Biosystems automatic sequencer. Chromatograms were assembled and initial base calls were made for each gene with Phred (Green and Ewing 2002) and Phrap (Green 1999) as orchestrated by Mesquite Ver. 3.4 (Maddison and Maddison 2018) and Chromaseq vers. 1.3 (Maddison and Maddison 2017). Final base calls were made in Mesquite and ambiguous bases were designated by a standard ambiguity code. Resulting sequences were deposited in GenBank (Table 1). Previously published COI sequences of *Hypoprepia* and all other members of the tribe Lithosiini were downloaded from GenBank and the Barcode of Life Database (Table 1). All 500 sequences were assembled into a single matrix and were aligned using MAFFT vers. 7 (Katoh and Standley 2013). The aligned matrix was partitioned by codon position, with each codon position allowed to have independent parameter values for the model of evolution. Maximum likelihood (ML) heuristic searches were conducted using RAxML 8.0.9 (Stamatakis 2014) under the GTR+gamma model of evolution on CIPRES Science Gateway portal (Miller et al. 2010). 500 search replicates were conducted to find the maximum likelihood tree.

We identified the closest relatives of *Hypoprepia* in the resulting maximum likelihood tree, selected these as our outgroup taxa, and re-ran the ML heuristic searches (as described above) on the smaller matrix of 73 taxa. Clade support was conducted using rapid bootstrapping with a subsequent ML search and letting RAxML halt bootstrapping automatically (using MRE-based bootstopping criterion).

**Table 1.** GenBank/BOLD accession number of the species.

Species name	GenBank/BOLD Accession Number
**Outgroup**	
* Abrochocis esperanza *	KC571047.1
* Balbura dorsisigna *	KC571053.1
* Balbura intervenata *	KC571052.1
* Chrysochlorosia magnifica *	KC571057.1
* Cisthene angelus *	BBLOE1648-12
* Cisthene barnesii *	ABLCW009-10
* Cisthene barnesii *	LMEM919-09
* Cisthene barnesii *	RDNMF900-08
* Cisthene deserta *	ABLCW126-10
* Cisthene dorsimacula *	RDNMF903-08
* Cisthene faustinula *	LOCBC003-06
* Cisthene juanita *	IAWL658-09
* Cisthene kentuckiensis *	HKONS224-08
* Cisthene liberomacula *	LOCBC697-06
* Cisthene martini *	LMEM065-09
* Cisthene packardii *	LSUSA097-06
* Cisthene perrosea *	ABLCW128-10
* Cisthene picta *	LPOKA060-08
* Cisthene plumbea *	KC571059.1
* Cisthene polyzona *	BLPDD935-09
*Cisthene* sp.	LPYPC028-08
*Cisthene* sp.	LPYPC119-08
* Cisthene subjecta *	HKONS229-08
* Cisthene subrufa *	LPYPB681-08
* Cisthene subrufa *	LPYPC078-08
* Cisthene tenuifascia *	BBLSW086-09
* Cisthene unifascia *	ABLCW140-10
* Dolichesia falsimonia *	KC571062.1
* Gardinia anopla *	KC571075.1
* Lycomorphodes correbioides *	KC571088.1
* Lycomorphodes sordida *	KC571089.1
* Talara cara *	KC571098.1
* Talara lepida *	KC571099.1
* Talara nr. mona *	KC571100.1
**Ingroup**	
* Hypoprepia cadaverosa *	KC571080.1
* Hypoprepia cadaverosa *	MF922743.1
* Hypoprepia cadaverosa *	MF923063.1
* Hypoprepia cadaverosa *	MF923535.1
* Hypoprepia cadaverosa *	MF923758.1
* Hypoprepia cadaverosa *	MF923893.1
* Hypoprepia cadaverosa *	MF924076.1
* Hypoprepia fucosa *	MF923771.1
* Hypoprepia fucosa *	MF924037.1
* Hypoprepia fucosa *	KC571078.1
* Hypoprepia fucosa tricolor *	KC571079.1
* Hypoprepia inculta *	ABLCW242-10
* Hypoprepia inculta *	CMAZA783-10
*Hypoprepiainculta* 4170	**MH337839**
* Hypoprepia inculta *	RDNMG037-08
*Hypoprepiainculta* 3259	**MH337840**
* Hypoprepia inculta *	ABLCW240-10
* Hypoprepia inculta *	ABLCW241-10
* Hypoprepia inculta *	ABLCW244-10
* Hypoprepia inculta *	ABLCW245-10
* Hypoprepia inculta *	RDNME352-07
* Hypoprepia inculta *	MF923496.1
*Hypoprepiainculta* 3573	**MH337833**
*Hypoprepiainculta* 3574	**MH337841**
* Hypoprepia inculta *	ABLCW071-10
* Hypoprepia inculta *	ABLCW056-10
* Hypoprepia inculta *	ABLCW055-10
*Hypoprepialampyroides* sp. n. 3566	**MH337834**
*Hypoprepialampyroides* sp. n. 3567	**MH337835**
*Hypoprepialampyroides* sp. n. 3568	**MH337836**
*Hypoprepialampyroides* sp. n. 3569	**MH337837**
*Hypoprepialampyroides* sp. n. 3570	**MH337838**
* Hypoprepia miniata *	*BBLOB1474-11*
* Hypoprepia miniata *	*LBCC462-05*
* Hypoprepia miniata *	*LBCC769-05*
* Hypoprepia miniata *	*LGSMB301-05*
* Hypoprepia miniata *	*LGSMB302-05*
* Hypoprepia miniata *	*LOFLB682-06*
* Hypoprepia miniata *	*LOFLC311-06*
*Hypoprepia* sp.	*KT706007.1*

### Taxonomic treatment

Genitalic preparations were made following the methods of Jaeger (2017) by staff at the CNC. Genitalia were slide-mounted using Euparal and photographed with a Leica DFC450 camera, Leica Application Suite 4.8 with a Leica M205C stereo microscope, and processed in Adobe Photoshop. Photographs of the pinned adult male and female paratypes were made using Visionary Digital Imaging System with a Canon EOS 7D digital camera and Canon MP-E65mm f/2.8 1–5× lens. Multiple images were combined using Zerene Stacker version 1.04.

Repository abbreviations are as follows:

**CNC** Canadian National Collection of Insects, Arachnids and Nematodes, Ottawa, ON

**USNM** National Museum of Natural History (formerly United States National Museum), Washington, DC

**UAIC** University of Arizona Insect Collection, Tucson, AZ

**UNAM** Universidad Nacional Autonoma de Mexico, Mexico, DF

**DEBC** Don E. Bowman Collection, Golden, Colorado

**JDPC** John D. Palting Collection, Tucson, AZ

**RBNC** Ray B. Nagle Collection, Tucson, AZ

## Results and discussion

### Phylogenetic analysis

Our molecular phylogenetic analyses reveal strong support for the monophyly of *Hypoprepia* and a close relationship between *H.inculta* and *H.lampyroides* (Figure 15). It is noteworthy that *H.lampyroides* is recovered as a single well-supported clade. However, recognizing this clade as a new species renders *H.inculta* paraphyletic in the COI gene tree. Focusing on gene tree topology alone, one might decide not to recognize *H.lampyroides* as a new species, but rather view it as a unique population of *H.inculta*. However, we contend that these are two valid species since specimens of both occur in strict sympatry at the Rose Peak locality and they are easy to distinguish morphologically by size, wing color, antennal structure, as well as the form of both male and female genitalia. We predict that the 657 base pair fragment of COI does not contain enough phylogenetic information to infer the *Hypoprepia* species tree with accuracy. This is a common result of phylogenetic analyses of the COI barcoding region within some Lepidoptera (Beltran et al. 2002, Wiemers and Fiedler 2007) and within noctuoids in particular (Schmidt and Sperling 2008, Zahiri et al. 2017). The lack of reciprocal monophyly among species in the tree could also result from ongoing hybridization events resulting in mtDNA introgression, and/or incomplete lineage sorting (Funk and Omland 2003).

The phylogeny also suggests that *Hypoprepia* is in need of further revisionary work, particularly with respect to species boundaries between *H.miniata* and *H.cadaverosa*. These fully allopatric species (*H.miniata* common in the eastern US and *H.cadaverosa* common in the western US) look quite different from one another. Even so, several authors have suggested that they should be synonymized (Zahiri et al. 2017, Powell and Opler 2009). Given this and that both nominate forms are polyphyletic in our tree, it seems likely that these forms represent regional variation in the same species. Future investigations comparing their anatomy and phylogenetic analysis of additional genes, particularly nuclear genes, will help resolve this taxonomic question.

### Taxonomic treatment

#### Hypoprepia lampyroides

Taxon classificationAnimaliaLepidopteraArctiidae

Palting & Ferguson sp. n.

http://zoobank.org/746F6BFE-47B9-4E47-832B-F75A954A75C2

Figs 1–2 , 3–4 , 5 , 8 , 9–10 , 13–14 , 18 , 19

##### Type material.

Holotype ♂. Arizona:, [Apache Co.], White Mountains, Greer, 8,200 ft., 4–5 July 1988, R.H. Leuschner [USNM]. Paratypes 32♂ 3♀. Arizona: Santa Cruz Co., 8.5 mi. SE of Patagonia, Harshaw Canyon, 4,850 ft., 24 July 1998, D.E. Bowman, 1♀ [DEBC]; 29♂ 2♀, Greenlee Co., Blue Ridge Primitive Wilderness, US Hwy 191, vicinity of Rose Peak, 33°26'N 109°22'W, 8084 ft., 19 June 2017 [specimens distributed between JDPC (8♂), UAIC (6♂), CNC (5♂ 1♀), USNM (8♂ 1♀), UNAM (2♂), and RBNC (1♀)]. Mexico: 10 mi. W. of El Salto, Durango, 9,000 ft, 13 June 1964, J.E.H. Martin, 1♂ [CNC]; 2♂, Sonora, Mesa del Campanero, Barranca El Salto, elevation 6561’, Municipio de Yecora, , 2 July 2013, J. Palting [JDPC, UNAM].

##### Etymology.

The specific epithet *lampyroides* means “like *Lampyra*” referring to this species’ remarkable mimicry of a sympatric lampyrid beetle species, as discussed below.

##### Diagnosis.

**Figures 7–8.**
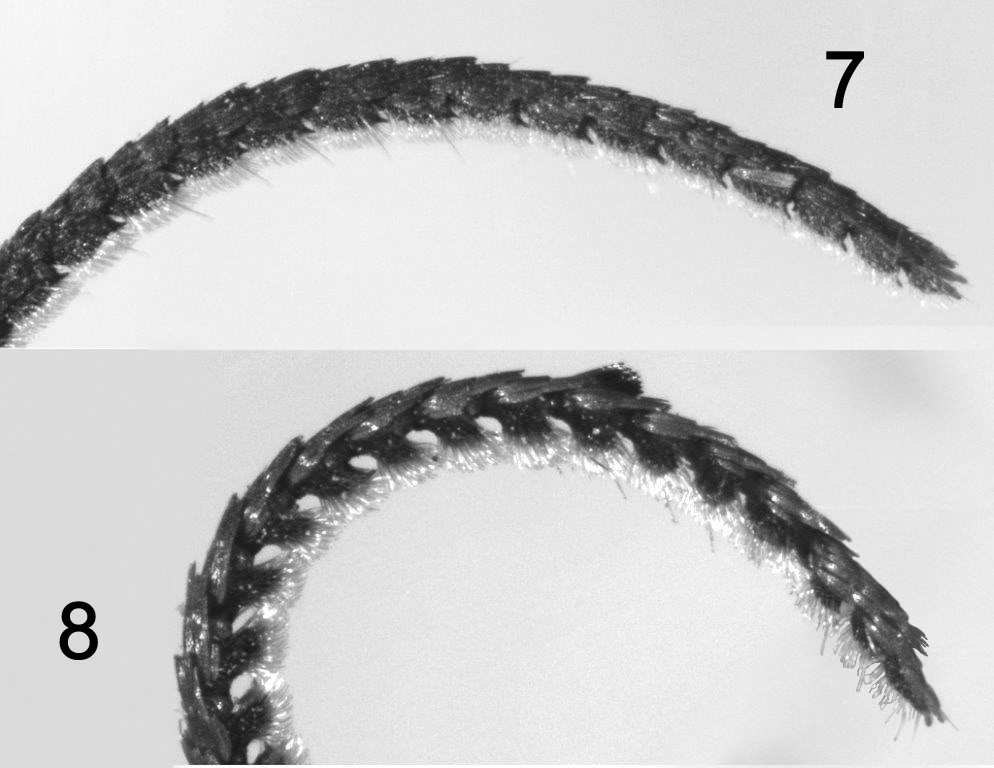
Lateral view of male antennae: **7***Hypoprepiainculta***8***Hypoprepialampyroides*

*Hypoprepialampyroides* (Figs 1–5) occurs sympatrically with *H.inculta* (Figure 6) and is easily distinguishable externally by its larger size; unmarked blackish forewings; brighter more extensively pink hindwings; somewhat different palpi; and different male antennae that more nearly resemble those of *H.cadaverosa.* The antenna differs structurally from that of *H.inculta* (Figure 7), which exhibit squarish, closely set segments (flagellomeres) with little space between them. The laminae of the antennal segments of *H.lampyroides* (Figure 8) are conspicuously raised, tapered, and appear farther apart when viewed laterally. The antenna of *H.lampyroides* is more like that of *H.cadaverosa*, a species that it does not otherwise resemble.

**Figures 9–12.**
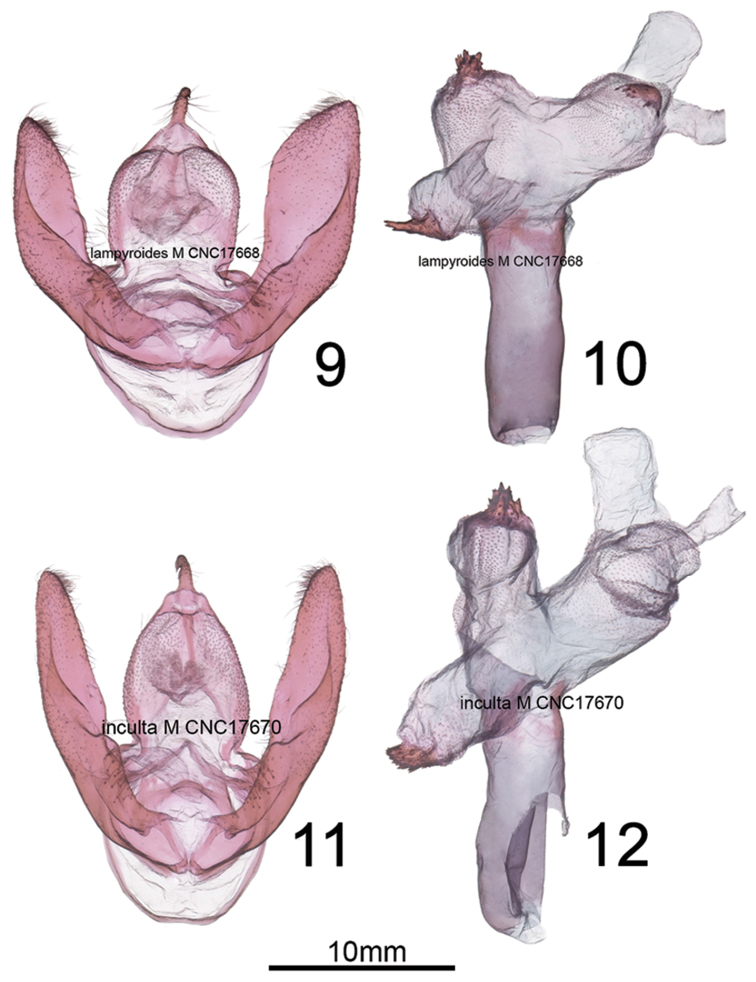
**9–10** Male genitalia of *Hypoprepialampyroides***11–12** Male genitalia of *Hypoprepiainculta*.

**Figures 13–14.**
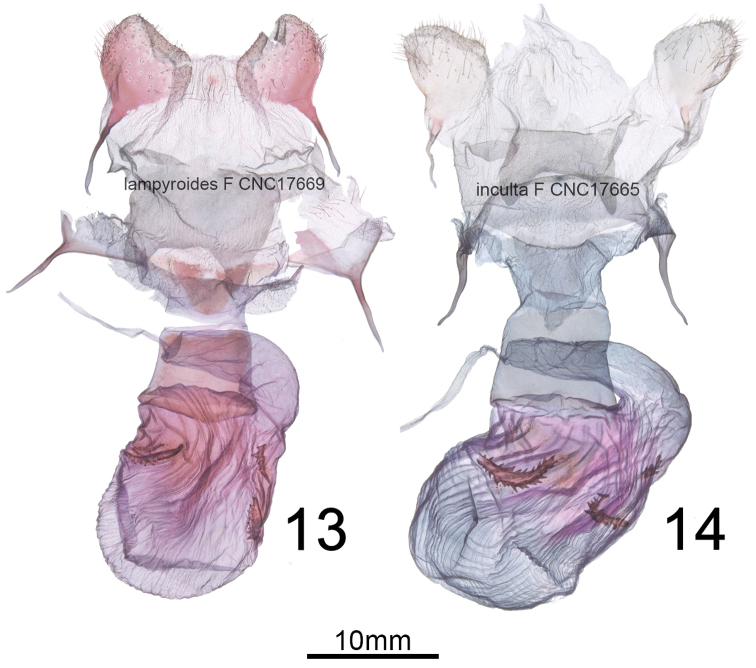
Female genitalia of **13***Hypoprepiainculta* and **14***Hypoprepialampyroides*.

Internally, the male *H.lampyroides* (Figs 9–10) differs from *H.inculta* (Figs 11–12) in the form of the spinose cornutus on the dorsal vesica chamber, which is apically elongated in *H.lampyroides* versus sawblade-like in *H.inculta*. *Hypoprepialampyroides* males always have three well-developed spinose cornuti (Figure 10), whereas the left ventrolateral cornutus (adjacent to the ductus) is often missing or reduced in *H.inculta* (Figure 12). The shape of the valve and tegumen is stouter and less elongate than in *H.inculta*. In females, the corpus bursae is globose (Figure 13) versus irregularly elongate in *H.inculta* (Figure 14), with four instead of three signa, the right-ventral signa possessing smaller spines than the corresponding right-ventral signa in *H.inculta*.

##### Description.

Sexes similar externally (Figs 3–4), but females with pink area on dorsal hindwing not quite as extensive, and with boundary between pink part and dark outer border more diffuse. ***Head.*** Vestiture of frons and vertex dark grey; labial palpus dark grey, upturned, slightly larger and longer than that of *H.inculta*, terminal (3^rd^) segment 1.25 × longer than 2^nd^; eye large, protuberant, more clearly exceeding a half sphere than those of the other *Hypoprepia* species; male antenna blackish, laminate, densely clothed with short setae beneath and with a few longer setae protruding sublaterally along the sides; female antenna simple, flagelliform.

***Thorax***. Dark brown or dark gray except for the tegula, which is mostly bright pink, matching basal spot of forewing; patagium blackish; legs entirely blackish or dark gray.

***Abdomen***. Vestiture gray, flushed with pink basally and terminally, ventrum entirely blackish or dark gray, except for some pink scales at distal end (*H.inculta* also may have a pink-tipped abdomen); ventral sternite A8 of males with reinforced, sclerotized rim-like anterior margin, but no pockets, coremata or androconial setae are visible on segments A7–A8. In females, pleurite of A7 with membranous but thick pockets, appearing somewhat rugose and more heavily sclerotized than surrounding integument. *Forewing.* Uniformly dark brown to charcoal gray, appearing blackish, unmarked except for a pink spot at base next to thorax, and lacking the pale streak on basal half of cubital vein seen in many *H.inculta*; male forewing length 17–20 mm, mean 17.5 mm (*n* = 6); female average forewing length 15.8 mm (*n* = 2) (usually 12–15 mm for both sexes of *H.inculta*). *Hindwing*. Hindwing pink, with a uniform, dark-gray costal and outer margin, ending just before anal angle; fringes gray to dark brown; ventrum of both wings similar to dorsum but slightly paler, and with more diffuse boundaries between pink and gray areas. *Male genitalia* (Figs 9–10) Generally similar to those of *H.inculta*; uncus cylindrical, flattened slightly laterally, oval in cross section, 8.8 × longer than wide; apex formed by slightly ventrally-curved, fine spine; basal two thirds with sparse, latero-basally directed setae; tegumen well-defined, rounded quadrate and dorsoventrally flattened with a slight constriction at juncture with vinculum; dorsal surface convex and bubble-like on either side of midline, densely covered in setal sockets distally; valve without clasper or process, slightly constricted basally, distal half rounded triangular, apex a rounded point, with short, broad somewhat spine-like setae along distal third of costal margin; sacculus not differentiated from remainder of valve, with a slight sub-basal, setose bulge; juxta indistinct, forming a dorsally emarginate rounded-rectangular transverse plate, approximately 4 × wider than long; phallus a straight, simple cylinder, 2.5 × longer than wide, coecum lacking; vesica consisting of three adjoining, globose chambers, the phallus appearing more or less as a tripartite club when vesica expanded; ventral chamber adjacent to ductus ejaculatorius, with additional lobe-like diverticulum, and with a spinose crest-like patch apically; laterodorsal chambers also with spinose crests. *Female genitalia*. (Figure 13) Papillae anales broadly diamond-shaped, sparsely setose; anterior and posterior apophysis relatively short, approximately equal in length to width of papillae; postvaginal aree with triangular scerlotization; ductus bursae short and broad, 1.5 × wider than long, highly flattened dorsoventrally and recurved ventrally; corpus bursae relatively small and globose, diameter 1.5–2 × width of ductus; signa consisting of two pairs of spinose straps, situated laterally near junction of ductus; cervix bursae situated right caudo-laterally and recurving left across ventral side of ductus.

##### Biology and distribution.

The brown eggs of *H.lampyroides* (Figure 16) were laid in small clusters inside a vial containing a piece of paper, and under magnification exhibit the “hammered copper” surface texture typical of lithosiine ova. These hatched after 14 days, the larvae being light yellowish initially then darkening as they fed. The larval stages are basically dark brown and unmarked throughout their development. Like other *Hypoprepia* (and other members of the subtribe Cisthenini) the larvae lack true verrucae (Bendib and Minet 1999) and instead have structures technically known as panniculae (Stehr 1987) with just one or two, stiff, black setae emerging from each (Figs 17–19). The larva is similar to *H.inculta*, which is also predominantly brown with black setae, while *H.cadaverosa*, reared by JDP at the same time as *H.lampyroides*, are marked with bright yellow bands (Figure 20). The larval mandible, dissected (Figure 21), shows the enlarged molar region found in other lithosiines. This feature has been suggested as a synapomorphy for the Lithosiini (Bendib and Minet 1999) and is believed to be related to their lichen diet. The larvae fed successfully on a mixed population of lichens obtained by shaving bark off oak trees, and developed through six instars into a caterpillar large enough to pupate. Unfortunately, lab conditions failed to yield a successful pupation, and the larvae eventually died. It is likely that *H.lampyroides* over-winter as a fully mature larva, pupating in the spring and emerging in early summer.

**Figure 15.**
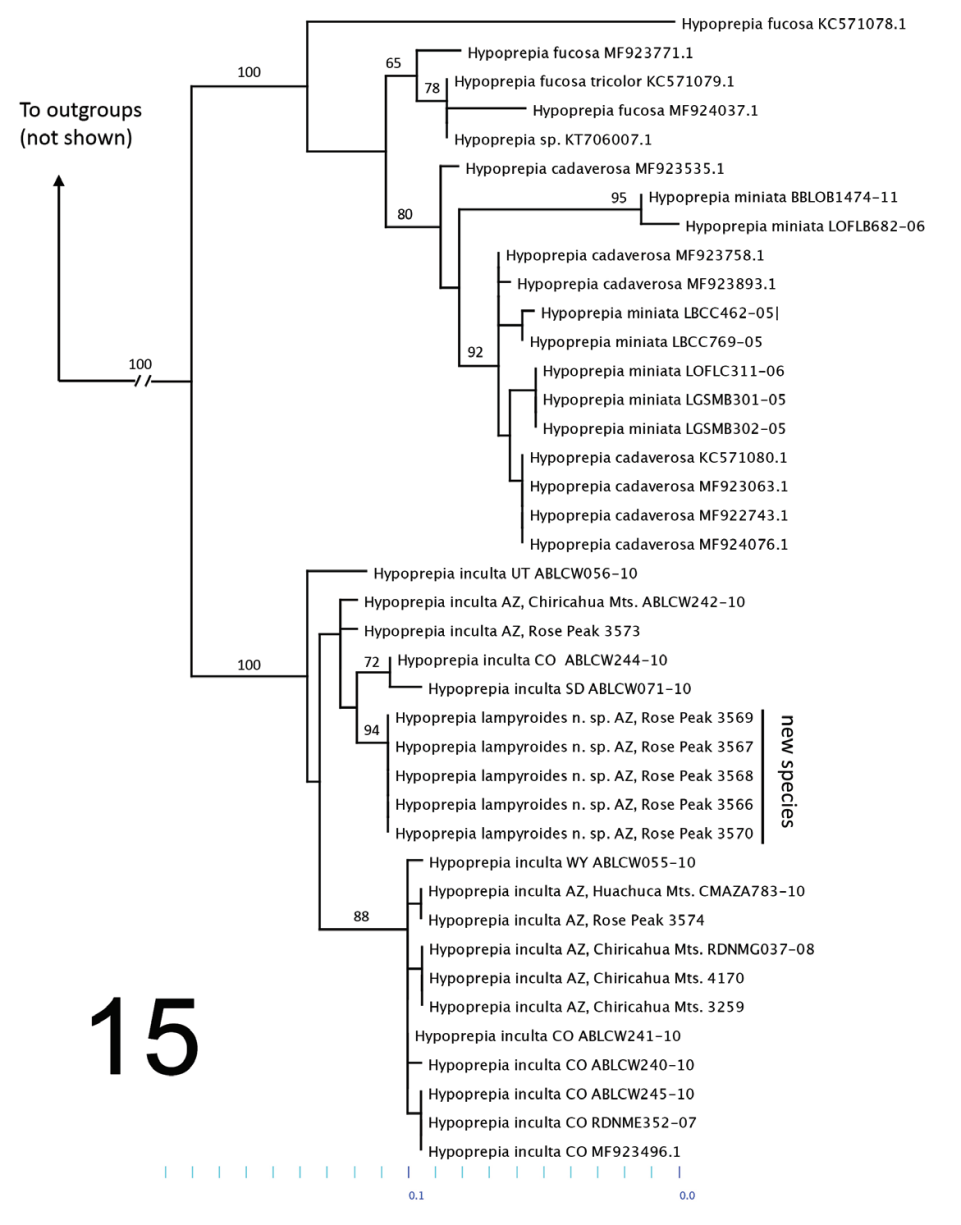
Maximum-likelihood tree of *Hypoprepia* species based on COI. Bootstrap values are reported on the branches subtending nodes with a support value greater than 50.

**Figure 16.**
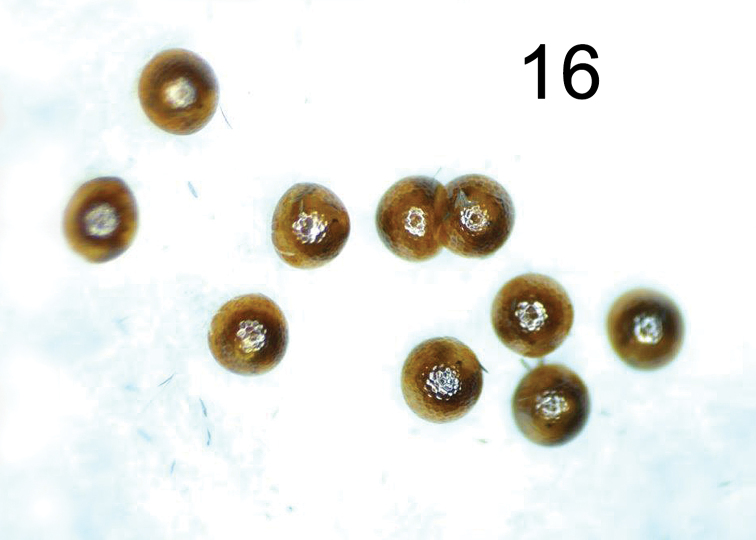
Eggs of *Hypoprepialampyroides*, approximately 20×.

The striking resemblance of this moth at rest (Figs 1–2) to a common southwest species of firefly, *Ellychniacorrusca* Linnaeus, 1767 (Coleoptera: Lampyridae) (Figure 23), points to them being part of a mimicry ring, which also includes another common montane beetle, *Discodonbipunctatum* Schaeffer, 1908 (Coleoptera: Cantharidae) (Figure 22). *Ellychniacorrusca* was common during the day in the Rose Peak area, and the bright pink markings on its pronotal region closely match the pink markings at the base of the forewing in *H.lampyroides*, likely affording the resting moths protection should a bird or other predator come upon them. Lampyrids are known to be chemically protected and distasteful to birds, but unlike most familiar nocturnal fireflies, *Ellychnia* lacks an abdominal light and is primarily diurnal. Research on sequestration of lichen polyphenolic compounds by other lithosiine arctiids (Hesbacher et al. 1995, Conner 2009, Scott et al. 2014) suggests that *H.lampyroides* itself has some chemical protection, thus the mimicry between these organisms is likely Mullerian. *H.inculta* is also likely part of this mimicry ring, although with its smaller size, dull pink markings, and grey wing color, it is a much less dramatic match to *Ellychnia* than *H.lampyroides*.

**Figures 17–18.**
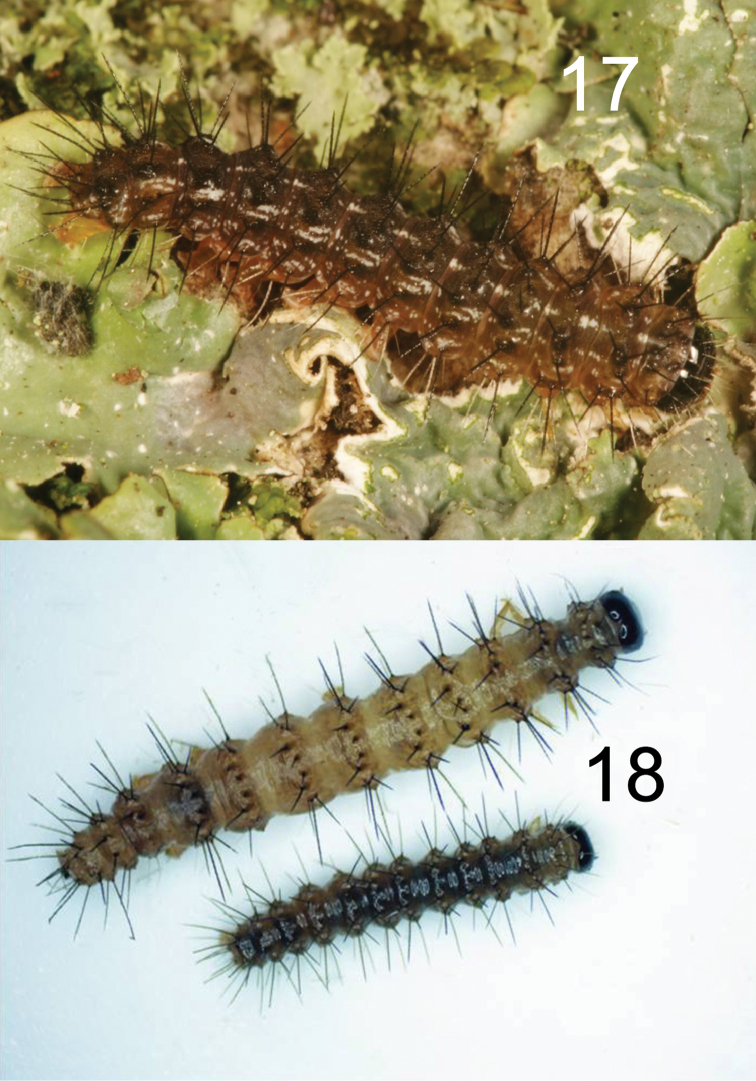
Larvae of *Hypoprepialampyroides*. **17** Living last instar larva and **18** Penultimate instar larvae, preserved.

**Figures 19–20.**
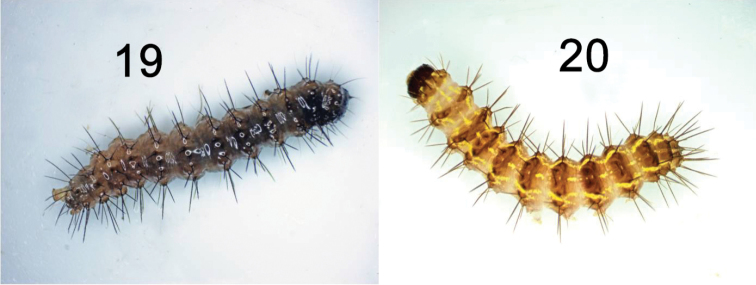
Last instar larvae of **19***Hypoprepialampyroides* and **20***Hypoprepiacadaverosa*, preserved.

**Figure 21.**
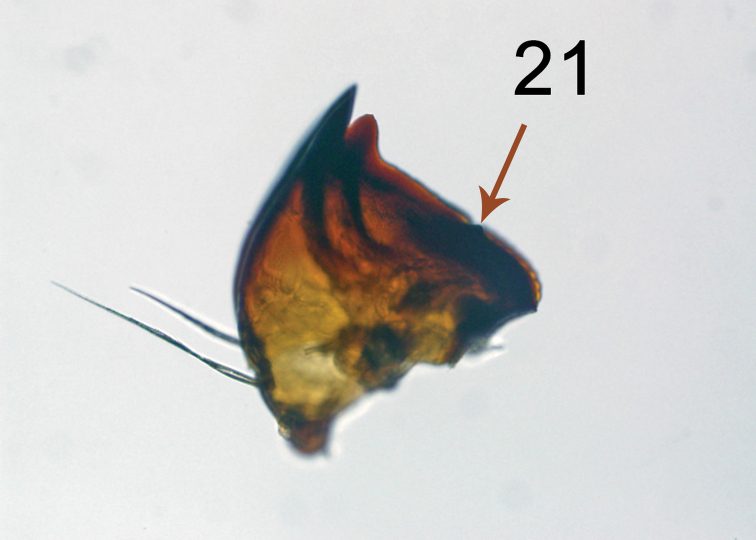
Mandible of last instar *Hypoprepialampyroides*, approximately 20×.

**Figures 22–23.**
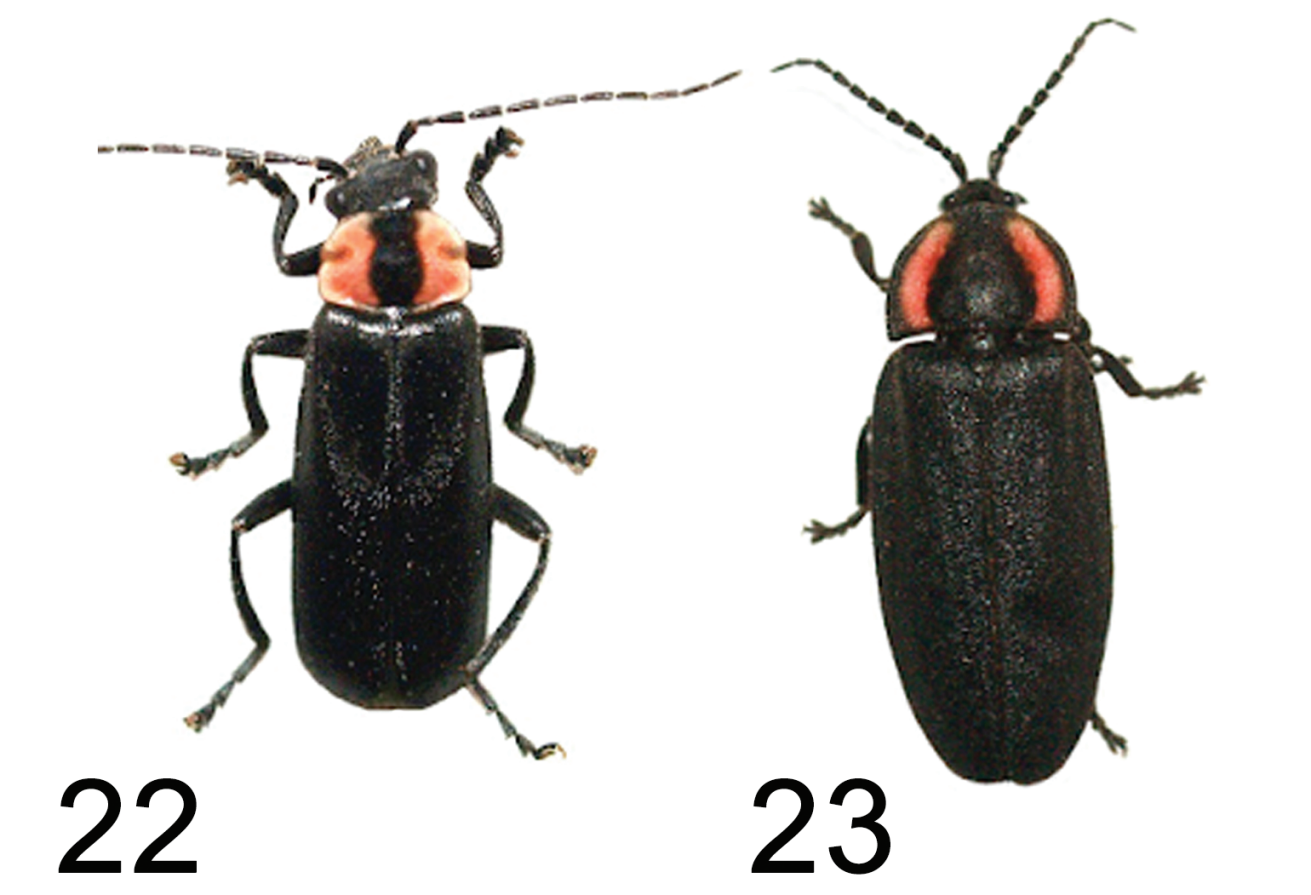
Mullerian mimicry with Coleoptera. **22***Discodonbipunctatum* (Cantharidae) **23***Ellychniacorrusca* (Lampyridae).

**Figure 24.**
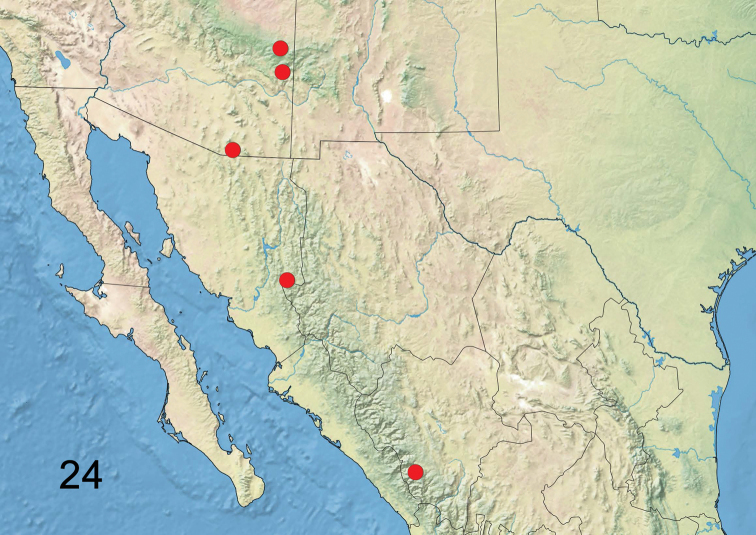
Range map of *Hypoprepialampyroides*.

*Hypoprepialampyroides* is known from over 30 specimens collected in Arizona, two specimens from Yecora, Sonora, Mexico and one from Durango, Mexico (Figure 24).

##### Remarks.

When examining the nearest relatives of *H.lampyroides*, Ferguson found that *H.inculta* from the southwestern United States is indistinguishable from the type material of *H.muelleri* Dyar, described from the vicinity of Mexico City, *H.muelleri* tends to have darker, more grayish hindwings, although in some *H.inculta* from Arizona they are equally grayish. Such a difference by itself is hardly significant. Unfortunately, fresh collected material of *H.muelleri* was not available for molecular analysis, but Ferguson’s conclusion based on his examination of the type material results in the following taxonomic change: *Hypoprepiamuelleri* Dyar, = *Hypoprepiainculta* Henry Edwards, syn. n. This extends the known range of *H.inculta* from as far north as Utah to the vicinity of Mexico City. *H.muelleri* had previously been the only member of the genus found exclusively in Mexico.

Ferguson found the Durango, Mexico specimen of *H.lampyroides* among unidentified arctiids from the Canadian National Collection. The region of El Salto, Durango, where it was collected, is mesic, conifer-dominated forest similar to that around Greer, Rose Peak, and Yecora, Sonora. The Harshaw specimen, a female, was collected by Don Bowman of Golden, Colorado and sent to Ferguson for identification. The Harshaw region is rather dry mid-elevation oak woodland/mesquite grassland, very unlike where all the other specimens of this moth have been collected.

## Supplementary Material

XML Treatment for Hypoprepia lampyroides
